# Landscape of Biomarkers and Actionable Gene Alterations in Adenocarcinoma of GEJ and Stomach—A Real World Data Analysis

**DOI:** 10.3390/cancers13174453

**Published:** 2021-09-03

**Authors:** Louisa Hempel, Julia Veloso de Oliveira, Andreas Gaumann, Valeria Milani, Katrin Schweneker, Kristina Schenck, Bastian Fleischmann, Patrick Philipp, Stefanie Mederle, Arun Garg, Armin Piehler, Beate Gandorfer, Cordula Schick, Axel Kleespies, Ludger Sellmann, Marius Bartels, Thorsten Oliver Goetze, Alexander Stein, Eray Goekkurt, Lucia Pfitzner, Sebastian Robert, Dirk Hempel

**Affiliations:** 1 Medical School, Sigmund Freud University, 1090 Vienna, Austria; louisa.hempel@med.sfu.ac.at; 2 Fraunhofer Institute of Optronics System Technologies, and Image Exploitation IOSB, 76131 Karlsruhe, Germany; julia.veloso@iosb.fraunhofer.de (J.V.d.O.); patrick.philipp@iosb.fraunhofer.de (P.P.); 3 Molekularpathologie Suedbayern, 87600 Kaufbeuren, Germany; Andreas.Gaumann@pathologie-kaufbeuren.de (A.G.); Lucia.Pfitzner@mpatho.de (L.P.); 4 Facharztzentrum Fuerstenfeldbruck, 82256 Fürstenfeldbruck, Germany; Valeria.milani@onko-medeor.de; 5 Oncological Center Dachau, 85221 Dachau, Germany; katrin.schweneker@onko-medeor.de (K.S.); kristina.riedmann@onko-medeor.de (K.S.); 6 Oncological Center Pfersee, 86157 Augsburg, Germany; bastian.fleischmann@onko-medeor.de; 7 Oncological Center Donauwörth, 86609 Donawörth, Germany; stefanie.mederle@innomedcare.de (S.M.); arun.garg@onko-medeor.de (A.G.); 8 MVZ Laboratory Freising, 85354 Freising, Germany; armin.piehler@onko-medeor.de (A.P.); beate.gandorfer@onko-medeor.de (B.G.); cordula.schick@onko-medeor.de (C.S.); 9 Cancer Center Dachau, 85221 Dachau, Germany; Axel.Kleespies@helios-gesundheit.de; 10 Praxis für Onkologie Moenchengladbach, 41066 Mönchengladbach, Germany; lsellmann@onko-mg.de (L.S.); bartels@onko-mg.de (M.B.); 11 Krankenhaus Nordwest IKF Frankfurt (Main), 60488 Frankfurt a.M., Germany; goetze.thorsten@khnw.de; 12 Hematology-Oncology Practice Eppendorf (HOPE) and University Cancer Center Hamburg (UCCH), 20144 Hamburg, Germany; stein@hope-hamburg.de (A.S.); goekkurt@hope-hamburg.de (E.G.); 13 Faculty of Applied Health and Social Sciences, Technical University of Applied Sciences Rosenheim, 83024 Rosenheim, Germany; sebastian.robert@th-rosenheim.de; 14 Institute of Translational Molecular Tumor Research, 85354 Freising, Germany

**Keywords:** GEJ, GC, next-generation sequencing, Her2neu, PD-L1, personalized medicine, molecular target, checkpoint inhibitors, actionable gene variants

## Abstract

**Simple Summary:**

Molecular tumor signatures are becoming increasingly important in the treatment of metastatic adenocarcinomas of the gastroesophageal junction (GEJ) and stomach (GC). There are few studies available analyzing data from the Caucasian population regarding molecular signatures and biomarkers. In the presented study, we investigated the distribution of gene variants in outpatients with advanced disease at the onset of diagnosis and correlated them with clinically relevant biomarkers according to ESCAT levels. In addition, we compared the results of conventional diagnostics (IHC/ISH) with NGS findings of gene amplifications. We were able to detect clinically relevant biomarkers according to ESCAT level I in approximately one-third of our patients, which have immediate therapeutic implications. The study highlights the importance of comprehensive molecular profiling for precision treatment of GEJ/GC and indicates that a biomarker evaluation should be performed for all patients with metastatic adenocarcinomas before the initiation of first-line treatment and during second-line or subsequent treatment.

**Abstract:**

After several years of negative phase III trials in gastric and esophageal cancer, a significant breakthrough in the treatment of metastatic adenocarcinomas of the gastroesophageal junction (GEJ) and stomach (GC) is now becoming evident with the emerging of precision oncology and implementation of molecular targets in tumor treatment. In addition, new generation studies such as umbrella and basket trials are focused on these molecular targets, which makes an early molecular diagnosis based on IHC/ISH and NGS necessary. The required companion diagnostics of Her2neu overamplification or PD-L1 expression is based on immunohistochemistry (IHC) or additionally in situ hybridization (ISH) in case of an IHC Her2neu score of 2+. However, there are investigator-dependent differences in the assessment of Her2neu amplification and different PD-L1 scoring systems obtained by IHC/ISH. The use of high-throughput technologies such as next-generation sequencing (NGS) holds the potential to standardize the analysis and thus make them more comparable. In the presented study, real-world multigene sequencing data of 72 Caucasian patients diagnosed with metastatic adenocarcinomas of GEJ and stomach were analyzed. In the clinical companion diagnostics, we found ESCAT level I molecular targets in one-third of our patients, which directly determined the therapy. In addition, we found potential targets in 14/72 patients (19.4%) who potentially qualify for precision therapies in corresponding molecular studies. The study highlights the importance of comprehensive molecular profiling for precision treatment of GEJ/GC and indicates that a biomarker evaluation should be performed for all patients with metastatic adenocarcinomas before the initiation of first-line treatment and during second-line or subsequent treatment.

## 1. Introduction

After a long period of therapeutic stagnation, a breakthrough in the treatment of metastatic adenocarcinomas of the gastroesophageal junction (GEJ) and stomach (GC) is now becoming evident with Her2neu, PDL1 or FGFR2 directed therapies [1]. The trastuzumab-deruxtecan conjugate led to a significant improvement of overall survival in patients with gastric cancer and ErbB2 overamplification after trastuzumab failure [2]. A second pillar is formed by PD-L1-directed ICI therapy, showing positive data for the first-line and even perioperative setting, recently presented at ASCO 2021. Based on phase III trials such as CheckMate 649, Checkmate 577, Attraction 4 and KEYNOTE 590 showing convincing clinical improvement, the addition of ICIs (nivolumab or pembrolizumab) to chemotherapy or chemoradiation therapy (CRT) is now becoming the standard of care in the first-line setting of metastatic disease [3, 4, 5, 6]. Another new treatment option is the addition of ICI therapy to Her2neu directed regimens in patients with ErbB2 overamplification. The first-time data from the Phase III KEYNOTE-811 trial, evaluating pembrolizumab in combination with trastuzumab plus chemotherapy for metastatic HER2+ gastric or GEJ adenocarcinomas, demonstrates a meaningful improvement in ORR compared with the standard combination of chemotherapy (74% vs. 52%, respectively; *p* = 0.00006) resulting in a Food and Drug Administration (FDA) approval for Her2+ patients [3]. A further therapy option is formed by the FGFR2 directed antibody treatments in combination with chemotherapy in patients with FGFR amplification. The FIGHT phase II study that evaluated the combination therapy of blemarituzumab and mFOLFOX6 showed an improvement in progression-free and overall survival in patients with FEGFR2 overexpression that was obtained in the IHC or gene amplification by ctDNA [4]. Beyond this, NGS enables the detection of therapy-relevant driver mutations, which, if actionable, represent the target structure for a specific treatment. NTRK fusion proteins represent an example of this therapeutic approach. If detected, the fusion protein can serve as a therapeutic target for larotrectinib or entrictinib [5]. Other experimental therapeutic targets such as c-Met, ErbB3 and mTOR are under clinical evaluation [6,7]. The therapeutical landscape of GEJ and GC is becoming increasingly complex. The identification of patients who qualify for targeted therapy based on appropriate biomarkers remains a major challenge.

## 2. Methods

The participating cancer centers had access to a hybrid capture-based NGS service platform (FoundationOne CDx, Grenzach-Wyhlen, Germany) for solid tumor samples and subsequently offered this service to patients with the advanced disease since April 2018 [8]. On 30 November 2017, the FoundationOne CDx test, based on an Illumina platform, was approved by the FDA to detect clinically relevant genomic alterations (point mutations, ins/dels, rearrangements and CNAs) and to support the selection of an appropriate personalized therapy by physicians [8]. The test was based on the examination of 324 genes, as well as introns of 34 genes involved in rearrangements. In addition, tumor mutation burden (TMB) and microsatellite instability (MSI) were evaluated [9,10]. Based on these results, a comprehensive molecular tumor profile was generated [11,12]. Hence, individual therapy options are suggested for tumor profiles according to the current state-of-the-art scientific knowledge and regulatory approval.

## 3. Patients’ Characteristics

All included patients had metastatic adenocarcinoma of the stomach or GEJ. A total of 72 patients were evaluated, 25.0% women and 75.0% men. Seventy-eight percent of patients received at least one line of therapy prior to molecular profiling, and almost one-third (22.0%) received more than two lines of therapy. Clinical characteristics of the enrolled patients are shown in Table 1.

PD-L1 status was determined using the Combined Positive Score (CPS), which was assessed with different antibody clones (SP263, SP142 and CAL10). Her2neu status was determined by immunohistochemistry (IHC) using DAKO antibody, and in case of an IHC score of 2+, an in situ hybridization (ISH) was performed. Assignment of genomic alterations to the corresponding signaling pathways was based on central tendency measures. An evaluation of the follow-up interval, which analyzes the clinical follow-up after a molecular-based treatment decision, is currently not available due to the short period of time and does not represent the primary endpoint of the study.

## 4. Results

**Figure 1:**
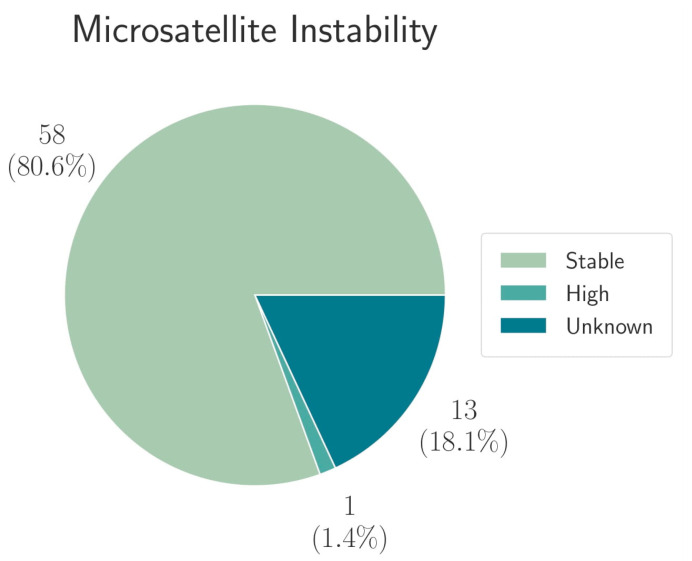
The distribution of microsatellite status among the 72 patients evaluated.

Regarding the treatment with ICIs, MSI and TMB and PDL1 CPS were analyzed as potential biomarkers. Based on IHC, the PD-L1 expression showing a CPS ≥ 5 was found in 9/72 patients (12.5%). Out of 72 patients evaluated, one (1.4%) patient was microsatellite instable (MSI), and 58/72 patients (80.6%) were microsatellite stable (MSS). In 13/72 (18.1%) patients, the evaluation was not possible (Figure 1).

**Figure 2:**
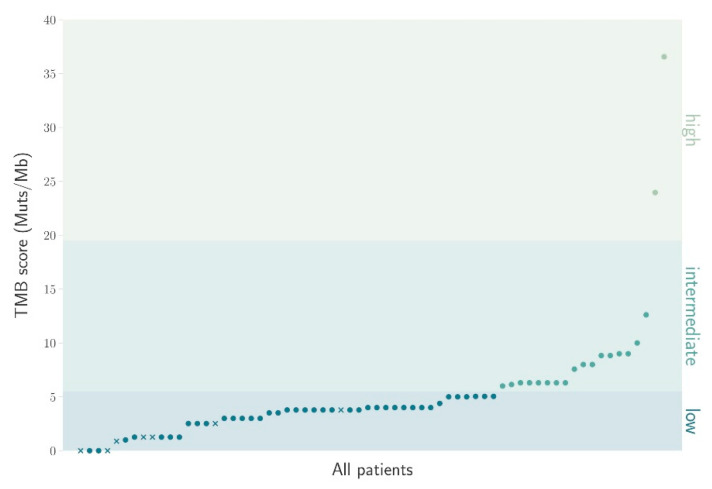
The distribution of patients according to TMB scores (*n* = 64). The TMB scores were divided into low, intermediate and high. The classification is based on mutations/mega base according to the FMI CDx test classification.

The evaluation of the TMB score showed that 3.1% (2/64) of the patients were found to have a high TMB score according to the FMI CDx test scale, while 70.3% (45/64) of the patients presented with a low TMB score. Out of the tested individuals, 26.6% (17/64) showed an intermediate score (Figure 2). The TMB score was available for 64 of the 72 enrolled patients.

**Figure 3:**
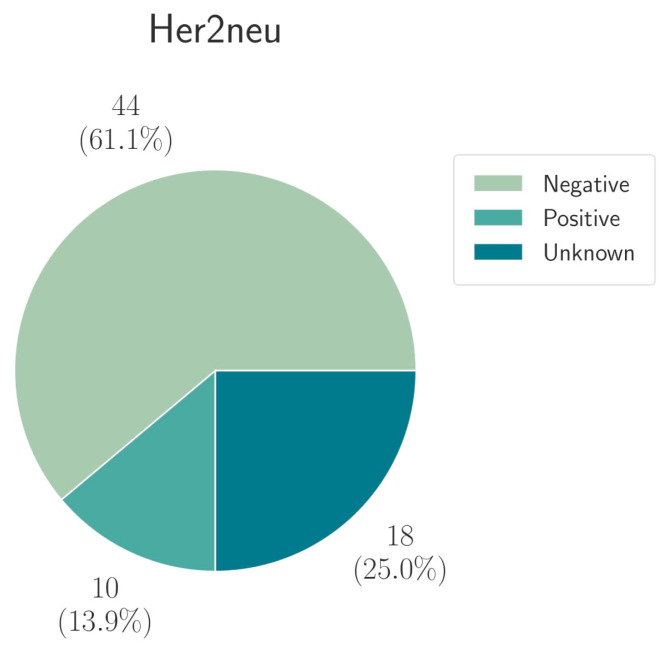
The proportion of Her2neu tested patients was divided into positive (IHC 3+ and IHC 2 with overamplification in ISH), negative (IHC 0; 1 and 2+ if ISH shows no overamplification) and unknown if no result was obtained.

A conventional IHC/ISH Her2neu status was available for 54/72 (75.0%) patients (Figure 3). A number of 10/54 (18.5%) patients had a positive Her2neu status in conventional diagnostics with IHC/ISH (IHC score 3+ or overamplification in ISH). Multigene sequencing analysis for Her2neu was available for all 54 patients. Sequencing analysis revealed an amplification of the ErbB2 gene in 8/54 patients (14.8%). Among these eight patients, two were Her2neu negative in conventional diagnostics (25.0%). Forty-six (85.2%) out of the 54 analyzed patients did not show amplification of the ErbB2 gene. Of these 46 patients, four were Her2neu positive (8.7%) in conventional diagnostics (Table 2).

**Figure 4:**
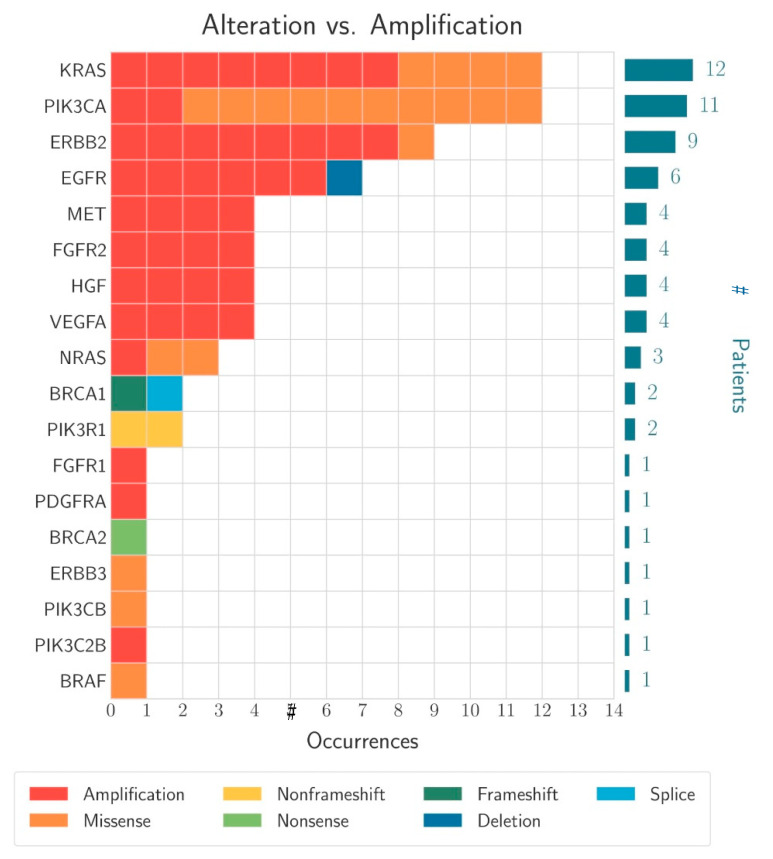
The different alterations in the affected genes that could be detected in the surveyed population (# indicates number of patients).

**Figure 5:**
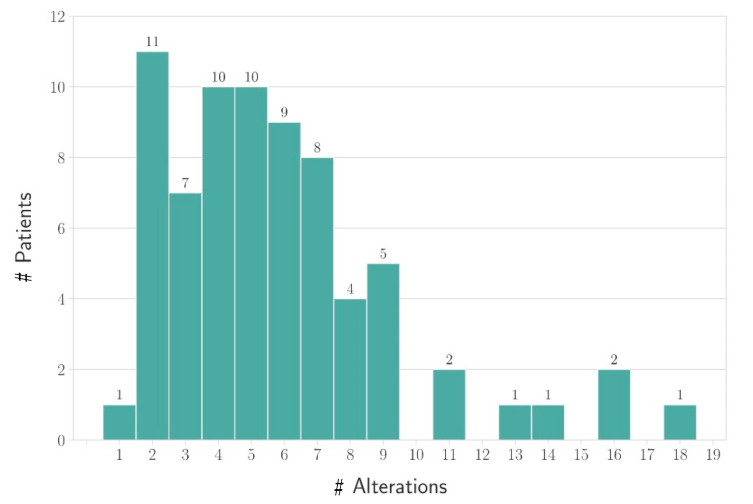
The number of detected alterations per patient (# indicates number of alterations).

**Figure 6:**
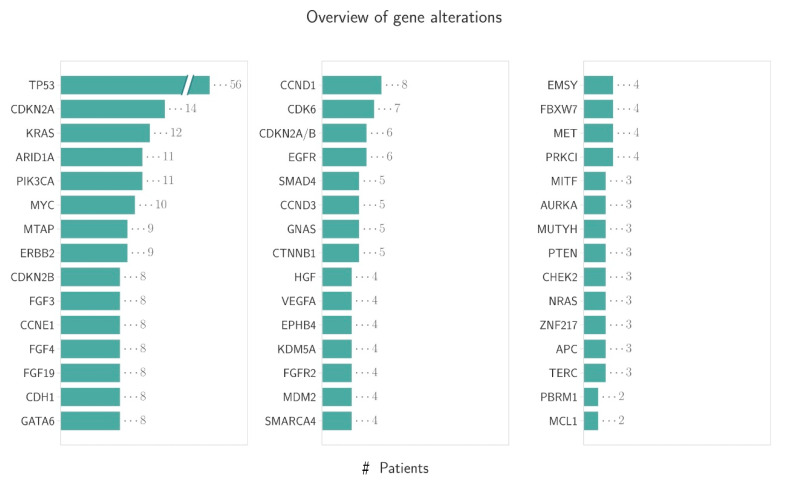
All detected gene alterations (# indicates number of patients).

The most frequent gene variations were detected in the KRAS gene (12/72; 16.4%), followed by PIK3CA mutations (11/72; 15.3%) as well as ErbB2 amplification (9/72; 12.5%), EGFR (6/72; 8.3%), VEGFA (4/72: 5.6%), FGFR2 (4/72: 5.6%) amplification and BRCA1/2 (3/72: 4.6%) mutations (Figure 4). On average, the patients were found to have alterations in 5.83 genes (Figure 5). An overview of all detected gene alterations is illustrated in Figure 6.

**Figure 7:**
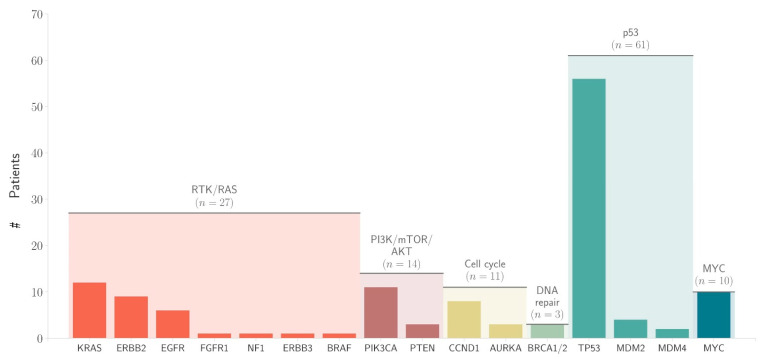
The alterations of the different genes and associated pathways (# indicates number of patients).

The most frequently altered oncogenic signaling pathway in our cohort was RTK/RAS (37.5%), followed by PI3K/mTOR/AKT (19.4%). The results of the pathway analysis are shown in Figure 7.

The detected gene variants can be classified according to their therapeutic relevance within the ESMO scale according to the level of evidence [13]. Table 3 and Figure 4 show the therapeutically relevant gene alterations in the patient population. Compared with the ESMO data, the distributions of gene alterations were within the expected range [13].

In Summary, we detected ESCAT level I biomarkers in 26/72 patients (36.1%). These were distributed among ErbB2 amplification (10/72), MSI/TMBhigh (3/72), CPS ≥ 5 (9/72) and FGFR2 amplification (4/72). In all patients with ErB2 amplification, detection led to treatment with trastuzumab (10/10) in addition to chemotherapy, while 8/12 patients with MSI/TMB high status or CPS ≥ 5 received a combination of chemotherapy and ICI.

## 5. Discussion

Metastatic adenocarcinomas of the gastroesophageal junction (GEJ) and stomach (GC) show marked intertumoral genetic heterogeneity. Thus, especially in the Asian region (Korea and Japan), where gastric carcinomas have the highest prevalence, early genomic sequencing is common [15,16]. There are few studies on the distribution of gene variants in the Caucasian population [14]. Increasing importance is given to next-generation sequencing and biomarker-driven therapy due to the possibility of appropriate targeted therapies. The European Society of Oncology (ESMO) recommends the use of NGS when biomarker-driven studies are available [17]. Early molecular analyses should be performed by combined use of NGS and IHC/ISH, as we were able to detect ESCAT stage I biomarkers in 36.1% of our patients. The biomarker analysis revealed the following distribution: ErbB2 amplification (10/72), MSI/TMB high (3/72), CPS ≥ 5 (9/72) and FGFR2 amplification (4/72). These results led to immediate clinical recommendations for therapy. Ten out of ten patients with ErbB2 over-amplification were treated with trastuzumab in addition to chemotherapy, and 9/12 patients who qualified for ICI therapy based on biomarkers received nivolumab or pembrolizumab in addition to chemotherapy. In our study, ErbB2 amplification is the most common clinically relevant biomarker with level I evidence. Clinical relevance is derived from the results of the ToGA trial and the Destiny Gastric 01 study2. Adding trastuzumab to chemotherapy in the ToGA trial improved median overall survival by 13.8 months compared to 11.1 months in those who received chemotherapy alone (hazard ratio 0.74; 95% CI 0.60–0.91; *p* = 0.0046) [18]. With the availability of the drug conjugate trastuzumab-deruxtecan, a new compound is available for patients with Her2neu amplification who were pre-treated with trastuzumab [2]. The Destiny Gastric 01 study showed an improvement in overall survival (12.5 vs. 8.4 months) in the trastuzumab-deruxtecan-treated study arm, which led to FDA approval for pre-treated patients with progressive disease [2]. The drug conjugate is already implemented in the NCCN-guidelines algorithm as a preferred second or further line option in Her2neu positive patients [19].

We found a Her2neu amplification in 8/54 (14.8%) patients analyzed by NGS, while 10/54 (18.5%) showed overexpression in the conventional diagnostics (IHC/ISH). These results were in the expected range of 20%, which was previously reported by Nie et al. [7]. These patients qualified for Her2-directed therapies, as established by the above-cited studies. In conclusion, all ten patients with ErbB2 amplification were treated with trastuzmab.

In the comparison of both methods (IHC/ISH and NGS), ten patients showed ErbB2 amplification in IHC/ISH. However, sequencing identified two additional patients who showed ErbB2 amplification but were negative for Her2neu in the IHC. These patients might have been excluded for targeted therapy by conventional detection methods alone. On the other hand, four patients showed no ErbB2 amplification in the NGS analysis but were found to be positive for Her2neu overamplification in the IHC. These data suggest that both detection methods alone can lead to patients being incorrectly considered ineligible for therapy. Therefore, the clinical trials should also correlate NGS results and the clinical course of Her2neu-based therapies.

Another option in the treatment of GEJ tumors and tumors of the stomach with ErbB2 amplification is the combination of ICIs (pembrolizumab and trastuzumab) in addition to 5 fluorouracil/platinum-containing chemotherapy. The FDA approved this combination on 5 May 2021 based on the Keynote-811 trial that evaluated patients with advanced gastric or gastroesophageal junction (GEJ) adenocarcinoma and Her2neu amplification who did not previously receive systemic therapy for metastatic disease [3]. However, the value of predictive biomarkers for response to ICI (CPS, CD274 amplification, and MSI/TMB level) in this patient population is unclear since, in both therapy groups, most patients had a CPC score ≥ 1% (verum group 88% compared to 85% in the placebo group) [3].

Thus, biomarker testing (CPS, CD 274 amplification, TMB/MSI) will also become increasingly important for this therapeutic strategy.

Further investigations should clarify whether there is a correlation between the CPS level, CD274 amplification, TMB, MSI and the response to a trastuzumab/pembrolizumab combination therapy. For this purpose, gene sequencing, including CD274, would be useful in addition to conventional IHC.

A further argument, in favor of the early use of molecular diagnostic, results from the introduction of ICI and the approval of nivolumab and pembrolizumab by the FDA.

Data from the global CheckMate 649 trial led to FDA approval for nivolumab in addition to FOLFOX or CapeOx chemotherapy as a backbone. In the CheckMate 649, 1581 patients were randomized. The median overall survival was 14.4 months with nivolumab plus chemotherapy versus 11.1 months for chemotherapy in the PD-L1 CPS ≥ 5 population (hazard ratio [HR] = 0.71; *p* < 0.0001) [20]. However, the CheckMate 649 study shows that the all-comer cohort also responds to therapy with ICIs [20].

The results highlight the urgent need to establish further predictive biomarkers for response to ICI therapy. Analysis of our data, considering a CPS ≥ 5 and TMBhigh/MSI, revealed that twelve patients qualified for treatment with checkpoint inhibitors and chemotherapy. In the future, the value of CD274 amplification should be evaluated in this context. For this reason, future NGS panels should also investigate the amplification of CD274 as an associated PDL1 gene and a potential biomarker for ICI response [21,22].

In the context of companion diagnostics, complementing both detection methods (IHC/ISH and NGS) could lead to greater accuracy in the identification of eligible patients and minimize investigator-dependent bias. In addition, the correlation of ICI-relevant biomarkers (CPS, CD274 amplification, MSI/TMB) with the clinical course should be evaluated in further studies.

A further molecular target is FGFR2 [23]. At this year’s ASCO, an update of the Fight trial reported a significant improvement in median overall survival in patients with amplification of FGFR in unresectable advanced or metastatic adenocarcinoma of the gastroesophageal junction with the addition of bemarituzumab to FOLFOX68. Bemarituzumab is a therapeutic IgG1 antibody directed against fibroblast growth factor receptor (FGFR2b) [4]. The addition of the antibody to chemotherapy (FOLFOX 6) was tested in a double-blind, placebo-controlled randomized phase II trial in unresectable locally advanced or metastatic gastric cancer in patients whose tumors showed FGFR2b overexpression by IHC or who had FGFR2 amplification by circulating tumor DNA (ctDNA) evaluation [4]. An improvement in median overall survival of 19.2 months (95% CI: 13.6 not reached) was shown in the treated group compared to the placebo group [4]. In our patient cohort, the NGS analysis revealed an amplification of the FGFR2 receptor in 5.5%. These patients potentially qualify for treatment with bemarituzumab with a significant clinical benefit.

Another argument for the early application of NGS in advanced disease is the available new study designs that are molecularly driven (umbrella and basket trials), for example, the PANGEA trial [24]. Patients with MET amplification, EGFR expression and amplification, as well as FGFR2 amplification, can be included [25]. In our study, MET amplification was found in 6.9% (5/72) of patients. MET has already proven successful as a target in the treatment of non-small lung cancer [26]. The VIKTORY trial, a Korean study, reported MET amplification in 3% (20/715) of the patients. The different incidence of MET amplification can be explained by the different population characteristics (Caucasians versus Asians) [27]. In the VIKTORY trial, all the patients received biomarker-based treatment. The patients in the biomarker assigned group had significantly longer PFS, and the response rates were highest in the MET-amplified (treated with savolitinib) group. Given the current evidence on both incidence and response rates, MET may therefore be a new target in the treatment of gastric adenocarcinoma, and further studies are needed to determine its role.

Most molecular-based studies currently test for RTK amplification (ERB2, MET, EGFR and FGFR2), and in our study, the RTK/RAS pathway was the most frequently altered oncogenic pathway (37.5%), followed by the PI3K/mTOR/AKT pathway (19.4%).

In our cohort, we found 14 patients with potential gene variants qualifying for molecular-based studies. Six patients had an EGFR amplification, and four patients revealed a VEGF-A, HGF and MET amplification. Despite the small number of patients, 19% had potentially treatable targets.

In the future, circulating tumor DNA should be considered in addition to tissue-based NGS both in terms of tumor heterogeneity and therapy selection [28].

## 6. Conclusions

The detected gene variants of ESCAT level I (36.1%) had a direct impact on the treatment decision. This underlines the importance of molecular diagnostics at the earliest possible stage of diagnosis in patients with metastatic adenocarcinomas of the gastroesophageal junction (GEJ) and stomach (GC). In our view, all patients should be screened for molecular targets and potential biomarkers such as MSI/TMB, ErbB2, PDL1 (CPS)/CD274 amplification, FGFR2 dMMR and NTRK fusion before initiation of the first-line treatment. Other promising therapeutic targets for clinical trials that were identified in our study are the PI3K/Akt/mTOR pathway (potential drug of RADPAC trial: everolimus), c-MET gene variants (potential drug: c-MET inhibitor: tivantinib), EGFR family gene variants (ErbB-1/HER1, ErbB-2 (new, HER2), ErbB-3 (HER3) and ErbB-4 (HER4)). In the context of precision oncology, comprehensive molecular profiling strategies will be indispensable.

## Figures and Tables

**Table 1:** The patient’s characteristics.

Patients Characteristics	All Patients (*n* = 72)	Comorbidities/Comedications	All Patients (*n* = 72)
Age in years	62 (±14)	Comorbidities	
Sex		Hypertension	13 (18%)
		Diabetes	7 (10%)
Female	18 (25%)	Nicotine abuse	6 (8%)
Male	54 (75%)	Cerebral infarction, stroke	3 (4%)
Mortality		Myocardial infarction	3 (4%)
Survived	25 (35%)	Heart failure	3 (4%)
Died	19 (26%)	Hypercholesterolemia	1 (1%)
Unknown	28 (39%)	COPD	1 (1%)
Cancer type			
Stomach	43 (60%)	Comedecation	
Esophagus	29 (40%)	Ondansertron	40 (56%)
Disease type		Glucocorticoid	63 (87%)
M1	72 (100%)	Granisetron	32 (44%)
		Loperamid	43 (60%)
Cancer treatment		Pantoprazole	29 (40%)
Surgery	8 (11%)		
Padiotherapy	9 (12%)		
Chemotherapy			
Immunotherapy	10 (14%)		
Trastuzumab	8 (11%)		
ICI			
Ramucirumab	9 (12%)		

**Table 2:** Comparison of conventional Her2neu diagnosis based on IHC/ISH with results of NGS.

Her2neu Resultes		Sequencing (Amplification)		
		Yes	No	Total
Conventional (IHC/ISH)	Positive	6	4	10
	Negative	2	42	44
	Total	8	46	54

**Table 3:** The ESMO classification of the alterations of various genes and their therapeutic relevance by ESCAT level. Table adapted from [14].

Gene	Genomic Alteration	Prevalence	ESCAT
ERBB2	Amplification	11% (8/72)	IA
	Mutation	1.4% (1/72)	IIIA
MSI/TMB high		4.2% (3/72)	IC
EFGR	Amplification	8.3% (6/72)	IIB
MET	Amplification	6.9% (5/72)	IIB
FGFR2	Amplification	5.5% (4/72)	IC
BRCA1/2	Mutation	4.1% (3/72)	IIA

ESCAT level I, the match of an alteration and a drug was validated in clinical trials and should drive treatment decision in daily practice: IA prospective—randomized clinical trial(s) show in a specific tumor type, an improvement of a survival endpoint. IB prospective—non-randomized clinical trial shows that in a specific tumor type, a benefit is defined by ESMO. IC clinical trials across tumor types or basket trials show clinical benefit level II, a drug that matches the alteration has been associated with responses in phase I/II or in a retrospective analysis of randomized trials; level III—alterations that are validated. Another cancer but not in the disease to treat, and level IV includes hypothetically targetable alterations based on preclinical data [13].

## Data Availability

Data are available on request from the corresponding author.
